# Alternate delivery platforms and implementation models for bringing evidence-based behavioral interventions to scale for youth facing adversity: a case study in West Africa

**DOI:** 10.1186/s43058-022-00259-5

**Published:** 2022-02-16

**Authors:** Laura Bond, Jordan Farrar, Ryan C. Borg, Katrina Keegan, Katharine Journeay, Nathan Hansen, Emmanuel Mac-Boima, Alimamy Rassin, Theresa S. Betancourt

**Affiliations:** 1 Boston College School of Social Work, 140 Commonwealth Avenue, Chestnut Hill, MA 02467 USA; 2 University of Georgia College of Public Health, Health Sciences Campus, Athens, GA 30602 USA; 3 Caritas Sierra Leone, 19 Savage Street, Freetown, Sierra Leone

**Keywords:** Mental health, Low-resource setting, Conflict-affected regions, Collaborative team approach, Alternate delivery platforms, Sierra Leone, Evidence-based intervention, Scaling out, Youth, Employment/entrepreneurship programs

## Abstract

**Background:**

Youth Functioning and Organizational Success for West African Regional Development (Youth FORWARD) was launched as an implementation science collaboration focused on scaling out evidence-based mental health interventions for youth exposed to war and other adversities through novel delivery platforms. This implementation science case study examines the use of a collaborative team approach (CTA) as a scale-out strategy to foster the integration of an evidence-based group mental health intervention, the Youth Readiness Intervention, into youth employment programs tied to regional economic development in Sierra Leone.

**Methods:**

A case study methodology is used to explore the feasibility and acceptability of integrating an evidence-based intervention, the Youth Readiness Intervention (YRI), into youth entrepreneurship programs (ENTR) in Sierra Leone, facilitated by the CTA. The authors analyzed field notes logged during program implementation, 8 weeks of supervision notes, 20 interviews with agency leaders and front-line staff delivering the YRI within this alternate delivery platform. Quantitative dissemination and implementation interviews administered to youth, facilitators, and agency leaders were analyzed using descriptive statistics and mixed linear models. A linked Hybrid Type II effectiveness-implementation cluster randomized trial is evaluating the clinical effectiveness of the YRI within this delivery platform.

**Results:**

Extant data indicate the strong feasibility and acceptability of integrating the YRI into the ENTR program. Facilitators of integration of the YRI into the ENTR include mission alignment of the organizations with the delivery of psychosocial interventions, shared commitment to serving vulnerable youth, support from local District Youth Councils, and high interest from the youth served. Barriers include perceived competition between frontline organizations seeking funding for psychosocial interventions, and challenges in flexibility between donors and implementation partners operating in a fragile/post-conflict setting. The CTA was a feasible and acceptable strategy to support fidelity and quality improvement while scaling out the YRI.

**Conclusions:**

Youth entrepreneurship and livelihood programs offer a promising mechanism for expanding the reach of evidence-based interventions to youth in fragile and post-conflict settings. Quality improvement and sustainment of evidence-based interventions are novel concepts in such settings. The CTA strategy institutionalizes the integration of an evidence-based intervention into youth entrepreneurship programs.

**Trial registration:**

NCT03603613 (phase 1 pilot, registered May 18, 2018) and NCT03542500 (phase 2 scale-out study, registered May 18, 2018).

Contributions to the literature

- This case study supports the feasibility and acceptability of CTAs as a method to enhance supervision and fidelity monitoring in a low-resource setting with poor infrastructure. Stronger understanding and buy-in from agencies, donors, and policymakers in such regions is critical to advancing investments in mechanisms such as CTAs which can enhance quality and sustainment of evidence-based practices.
- This case study addresses the challenges inherent in the integration of evidence-based group mental health interventions into youth employment programs in fragile settings. Findings contribute to recognized gaps in the literature, including lack of implementation research in the context of humanitarian disasters and post-conflict settings.
- Data demonstrates the acceptability and feasibility of delivering an evidence-based intervention through alternative delivery platforms. The YRI is successful when delivered by lay workers, showing that front-line youth employment workers can be effective interventionists for EBIs when provided with thorough training and when supported with integrated supervision and fidelity monitoring approaches through a CTA context.

## Background

The risk of long-term mental health problems is increased in settings where exposure to war and community violence compound other social problems. Studies in post-conflict settings have shown that more than one in five people suffer from mental health disorders, and nearly one in ten have a moderate to severe mental health disorder at any point in time [1]. The Longitudinal Study of War-Affected Youth in Sierra Leone (LSWAY; 1R01HD073349-01), launched in 2002, found that youth exposed to war trauma demonstrated higher levels of psychosocial problems [2–4]. In particular, emotion regulation was identified as a major underlying mechanism that limited youth’s ability to participate successfully in opportunities such as employment and education programs. Adaptive and pro-social behaviors were associated with community acceptance and social support [5]. LSWAY findings informed the establishment of a research hub, Youth Functioning and Organizational Success for West African Regional Development (Youth FORWARD), which is an integrative psychosocial initiative developed to respond to the mental health and emotion regulation needs of youth in Sierra Leone through development initiatives such as youth employment programming.

Sierra Leone has limited healthcare infrastructure and faces challenges in the delivery of mental health services. An 11-year civil war (1991–2002), Ebola outbreak (2014–2015), recent mudslide (August 2017), and now the COVID-19 pandemic (2020) have devastated the healthcare system and exacerbated the mental health treatment gap. The Government of Sierra Leone is committed to meeting some of the core needs of the country’s youth and its economic and health policy agendas align to support advancement of mental health interventions for youth exposed to compound adversities. Recently, the Ministry of Health and Sanitation implemented a multi-level, collaborative approach to treat mental health holistically and incorporate the formal and informal structures that provide mental health treatment across the country [6]. Sierra Leone’s recent Economic Development plan includes policy actions to support youth entrepreneurship and skill development [7], and partnership with the World Bank prioritizes building human capital through education initiatives and job creation [8].

Given the current government’s prioritization of human capital formation, Youth FORWARD’s approach to integrated psychosocial programming is especially timely. Youth FORWARD has scaled out an evidence-based Youth Readiness Intervention (YRI) via an alternative delivery platform of a youth entrepreneurship program run by the German development agency, Gesellschaft für Internationale Zusammenarbeit (GIZ). GIZ’s multilevel model combines national-level policy and service sector approaches to support employment and income improvement for vulnerable, rural, and undereducated youth. Through a needs-oriented approach, GIZ’s Employment Promotion Programme ensures that young people gain qualifications for employment with training intended to strengthen labor market skills, increase income, and promote resilience to economic shocks [9, 10]. The YRI is a transdiagnostic intervention to assist youth facing complex problems using evidence-based best practices and underwent rigorous adaptation in-country to ensure cultural fit and appropriateness through the use of local parables, relevant language, and skills useful to the Sierra Leonean context [11]. It is designed to be delivered by lay workers in a group format, which deepens social connections and enables peer-to-peer support long after the intervention ends. The YRI incorporates evidence-based components from cognitive behavioral therapy: psychoeducation, relaxation techniques, assertive communication strategies, cognitive restructuring, behavioral activation, goal setting, and sequential problem solving [12]. The YRI was increased from 10 to 12 modules to respond to group needs and provide greater treatment for depression [13]. A randomized controlled trial of the YRI demonstrated that youth assigned to the intervention reported significantly greater improvements in emotion regulation and prosocial attitudes and behaviors compared to control youth and were six times more likely to persist in school [13].

In this study, the YRI was integrated into the entrepreneurship training program (ENTR) of GIZ’s employment platform using a Collaborate Team Approach (CTA). While the YRI addressed youth’s mental health, interpersonal, and emotional functioning, the ENTR prepared them for employment by providing skills training and mentorship around starting a sustainable income generating activity. By integrating the two programs, Youth FORWARD aimed to improve youth’s daily functioning and interpersonal relationships while simultaneously developing critical livelihood skills. A feasibility pilot was conducted in Kailahun district, followed by a rigorous scale-out study implemented in the Kono, Koinadugu, and Kailahun districts in Sierra Leone, where GIZ operates. We adhered to the Standards for Quality Improvement Reporting Excellence Implementation Studies (SQUIRE) in preparaton of this manuscript [14].

## Methods

### Design

This study assessed the feasibility and acceptability of integrating the YRI and ENTR, while using a CTA scale-out strategy. A Hybrid Type II Effectiveness-Implementation Cluster Randomized three-arm design allowed for the simultaneous study of implementation and clinical effectiveness [15]. Implementation outcomes included indicators of feasibility, acceptability, and fidelity as well as YRI facilitator and youth satisfaction. Effectiveness outcomes, which will be reported elsewhere, concerned impacts on youth emotion regulation, interpersonal functioning, and participation in livelihood generating activities (manuscript submitted for publication, Desrosiers et al.; manuscript in preparation, Akram et al.; manuscript in preparation, Freeman et al.).

### Framework

**Fig. 1:**
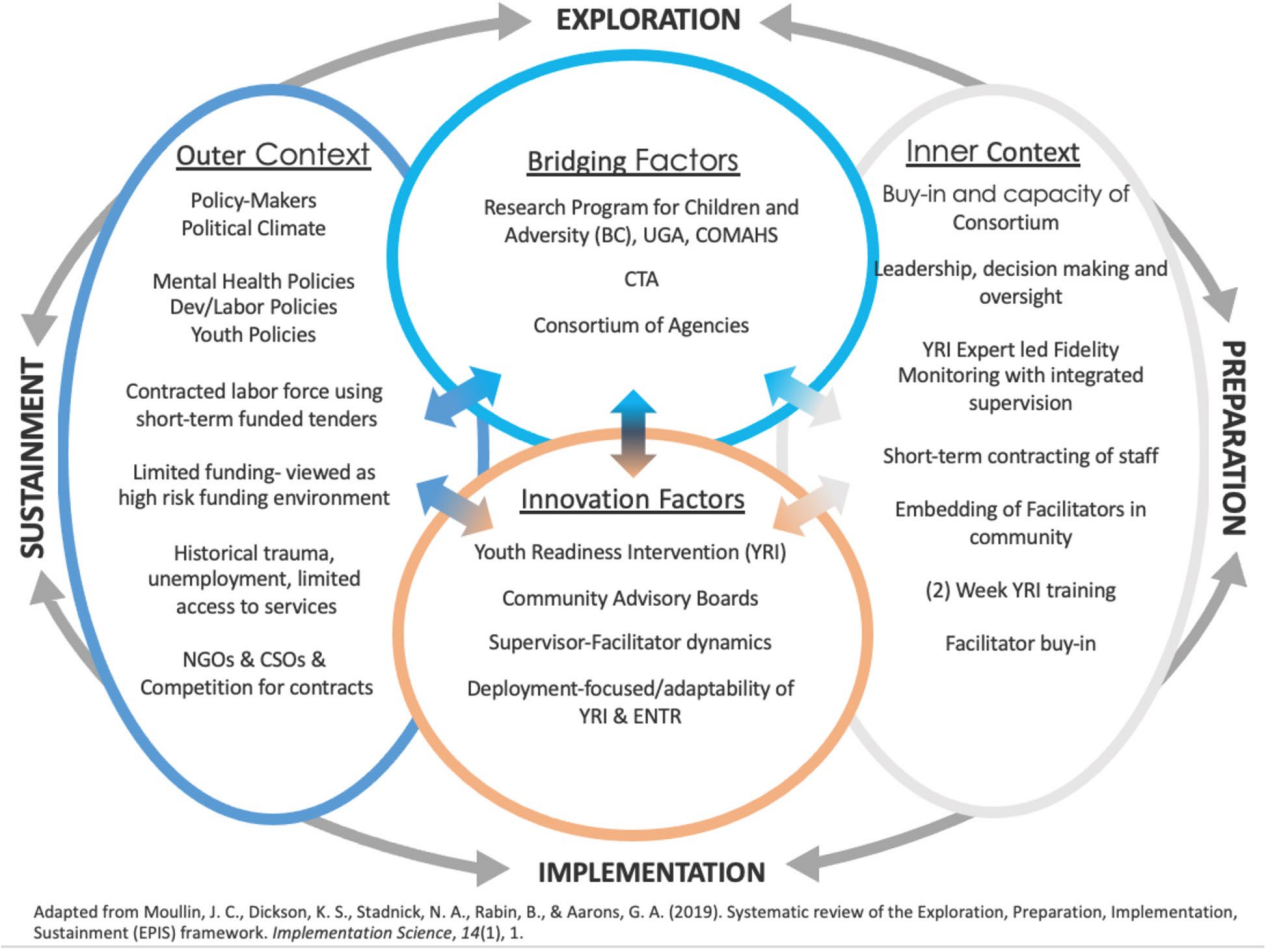
EPIS framework

**Fig. 2:**
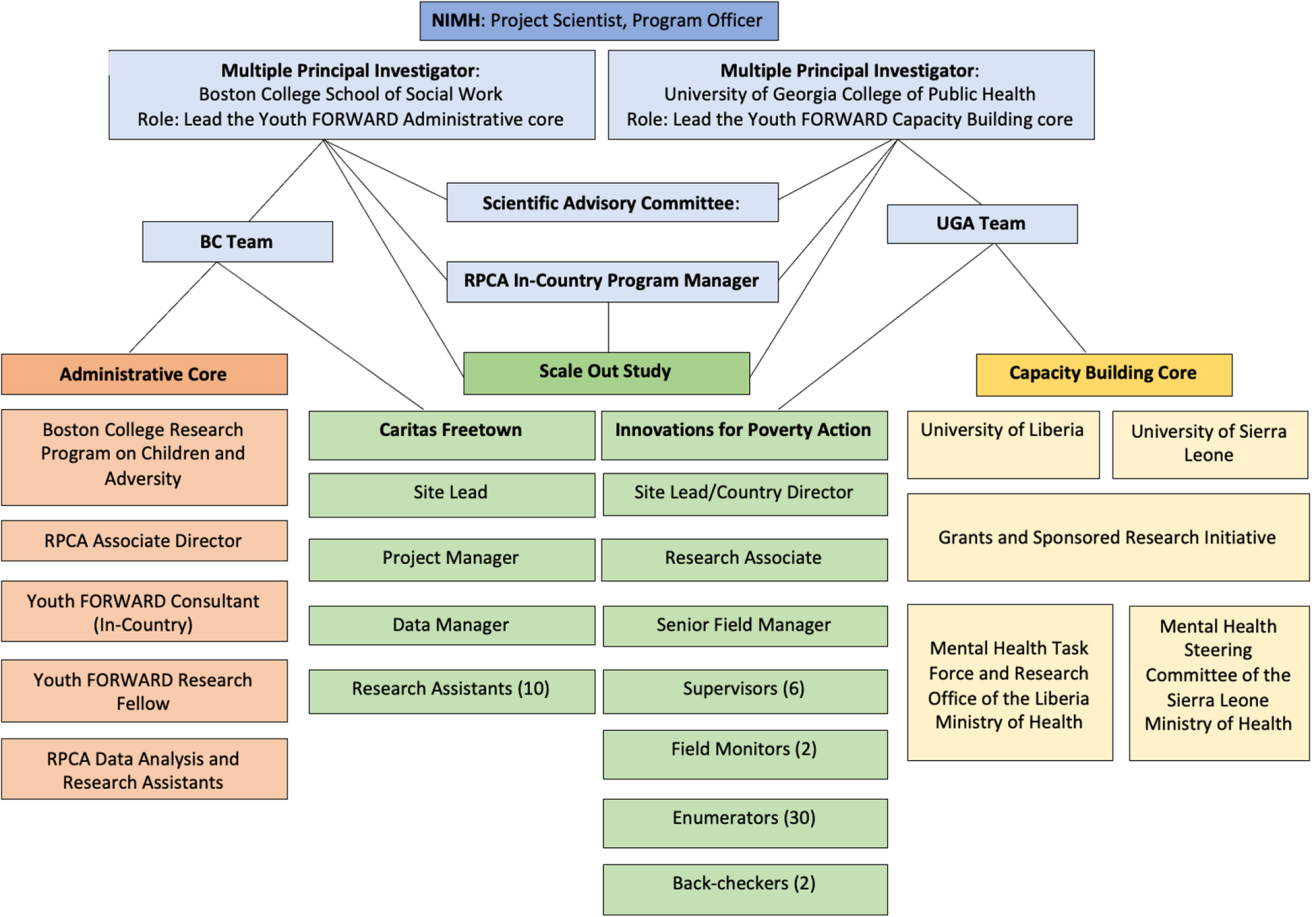
Youth FORWARD leadership structure

The Youth FORWARD CTA was intended to help address the issue of limited human resources by providing evidence-based mental health services while advancing goals shared with government and development actors (See Figs. 1 and 2). This approach increased opportunities for youth engagement in livelihood activities by training front-line lay workers to deliver the YRI to youth before participation in the ENTR.

The strategic use of the CTA was guided by the Exploration, Preparation, Implementation, and Sustainment (EPIS) framework, which allowed for scaling and sustaining the YRI and integrating it into the ENTR [16]. The EPIS framework accounts for the policy context within the country or at the local level as well as organizational service delivery conditions and has functioned well in health settings in low- and middle-income countries (LMIC). The EPIS framework is also helpful for assessing “fit” between an evidence-based practice and the intervention itself, considering innovation factors, bridging factors between the inner (organization) and outer (community) contexts, and the potential for sustainability of the intervention. For child and adolescent mental health interventions, bridging factors are particularly important because many services for youth facing adversity take place in public sector systems that span the social ecology [17].

The research team used the EPIS framework to develop an adapted model, demonstrating which factors were significant for the YRI+ENTR context (See Fig. 1). The framework was used to stage the project and assess progress while identifying challenges. Continuous quality improvement methods, such as integrated supervision and fidelity monitoring, Plan-Do-Study-Act (PDSA) cycles, and cross-site knowledge exchange, were utilized to ensure the YRI was delivered with fidelity and sites were able to benefit from lessons learned from other sites as they identified and addressed challenges [18]. The partnership between universities and community agencies served to bridge the inner and outer contexts in Sierra Leone. YRI+ENTR’s focus on deployment served as an innovation factor that mitigated outer context challenges such as historical trauma, unemployment, and limited access to services.

### Implementation approach

Implementation models which rely heavily upon remote expertise for training and fidelity monitoring are a major obstacle to achieving sustainable results as they fail to develop local expertise [19]. Therefore, Youth FORWARD’s pilot and scale-out study used a CTA to scale and sustain the YRI and integrate it into the ENTR in order to shift decision-making and ownership to community stakeholders. The CTA is modeled after the Interagency Collaborative Team model utilized in scaling up the SafeCare intervention for families involved in or at-risk for involvement in the child welfare system [20]. Through this work, collaboration emerged as a key element for the implementation and sustainment of evidence-based practices into established service delivery systems. The Interagency Collaborative Team model is contingent upon a multiagency commitment and partnership with a range of stakeholders from the onset [21]. The Youth FORWARD CTA included the YRI developers, YRI experts (members of the Caritas Freetown staff who were trained by the YRI developers and had prior experience implementing the YRI), YRI facilitators (local service providers contracted by GIZ to deliver the YRI and ENTR program), the Youth FORWARD research team, GIZ leadership, the contracted service provider agencies overseeing delivery of the YRI and ENTR program, and Caritas Freetown leadership. The CTA’s key activities included establishing a seed team, cross-site learning, collecting process data for monitoring quality and assessing barriers and facilitators, and communicating across stakeholders.

### The seed team

The CTA catalyzed system-wide YRI sustainment through development of a local core unit of experts—a seed team—to provide leadership and guidance on the delivery of the intervention. The function of a seed team is to draw from their experience and contextual knowledge to monitor, supervise, and mentor new facilitators as they deliver the intervention while overseeing cross-site collaboration to expand institutional knowledge on best practices in program delivery [21]. The seed team consisted of three YRI experts who provided ongoing training, coaching and support to YRI facilitators as the intervention moved from pilot to scale-out. Fidelity to evidence-based practices was achieved by meeting individually with YRI facilitators before each YRI session to ensure they were prepared and through weekly supervision meetings with YRI facilitators.

### Implementation development

An intensive 2-week training course that was initially designed by the YRI developers was updated by the Youth FORWARD seed team members for the pilot and scale-out studies. The seed team members delivered the course and prepared new YRI facilitators to deliver the intervention. During the training, facilitators received an intervention manual to help them learn the YRI and guide them in its delivery. To become a YRI facilitator, the trainees committed to participating in enhanced supervision and reporting processes, working across intervention sites, collecting process data throughout the intervention, and engaging in problem-solving to create a feedback loop allowing for quality improvement of intervention delivery over time.

### Scale out from feasibility pilot

The design of the YRI scale-out was first tested in a feasibility pilot from July through October 2018 in a rural district, Kailahun. In the pilot, youth were clustered and randomized to YRI+ENTR (*n* = 58) or ENTR-only (*n* = 57). A statistically matched comparison group (*n* = 60) was also recruited from Kailahun district. Due to logistical delays from the service provider, delivery of the YRI did not begin until the ENTR had completed, thereby undermining a study aim of investigating whether youth who received the YRI had better performance in the ENTR program. Findings from the pilot were further limited by a small sample size and non-randomized control group. However, the pilot allowed for pre-testing and refinement of study measures and testing of the CTA and communication structures when operating in rural Sierra Leone. In the scale-out study, modules were delivered in October and November of 2019 across 12 chiefdoms of Kailahun, Kono, and Koinadugu districts, with a total of 36 sites. The scale-out study increased engagement and collaborative problem solving with GIZ to ensure the YRI could be delivered before the ENTR and that adequate time was allowed for data collection given the larger sample size and expanded study area (See Table 1 for implementation and effectiveness study measures).

**Table 1:** Scale-out study measures

Outcomes	Time point	Instrument/psychometrics	Respondents
**Clinical outcomes** Emotion dysregulation, daily functioning, coping skills & prosocial attitudes, social support, intimate partner, relationships, anxiety, depression, stigma & risk, and behaviors	Quantitative ·Survey (baseline, post-YRI, post-ENTR)^a^ Qualitative ·Key informant interviews (baseline, post-ENTR)^a^ ·Focus groups^a^	· Difficulties in Emotion Regulation · WHO Disability Adjustment Scale · EQ 5 Health Questionnaire · Oxford Measure of Psychosocial Adjustment · Brief COPE scale · WHO Quality of Life-BREF · Responses to Stress Questionnaire · Revised Conflict Tactics Scale · Inventory of Socially Supportive Behaviors · Hopkins Symptom Checklist · Everyday Discrimination Scale · Adapted Youth Risk Behavior Survey · Post-Traumatic Stress Disorder Civilian Checklist · Daily Hardships · Goal Commitment Scale	Quantitative Youth (*n*=1151) Qualitative Key informants: Youth (*n*=90), YRI facilitators (*n*=12), agency leaders (*n*=2) Focus groups: Youth (*n*=40)
**Economic outcomes** Youth employment and economic self-sufficiency	Quantitative ·Survey (baseline, post-YRI, post-ENTR)^a^	· Income Generating Activities and Well-Being Measure	Youth (*n*=1151)
**Functional outcomes** Report on youth functioning and performance	Quantitative · Survey (baseline, post-ENTR)^a^	· Adapted Barkley Deficits in Executive Functioning Scale · Performance Survey adapted from classroom report used in prior YRI RCT · Teacher-Youth Rating Scale adapted from classroom report used in prior YRI RCT · Working and Training Performance Survey (self-created)	Third-party reporters (*n*=618)
**Implementation outcomes** Adoption, acceptability, appropriateness, feasibility, reach/access	Quantitative · Survey (baseline, post-ENTR)	· Applied Mental Health Research Implementation Science Measure	Youth (*n*=764), YRI facilitators (*n*=12), agency leaders (*n*=2)
**YRI fidelity**	Quantitative Administered for every YRI session	· YRI Fidelity Rating Guide	Filled out by a YRI expert

^a^Additional data collection planned for endline evaluation (rescheduled from 12 months post-ENTR to post-COVID-19)

GIZ utilized community sensitization and local media advertisements to recruit youth to participate in the ENTR. Interested youth submitted an application and were consented and screened for study eligibility by Caritas research assistants. Clusters of eligible youth, stratified by geographic location and gender, were randomized into one of three study arms: control (*n*=387), ENTR-only (*n*=380), and YRI+ENTR (*n*=384). Youth were quantitatively assessed at baseline and post-ENTR. In order to isolate YRI treatment effects and strengthen the YRI evidence base, a subsample of 396 treatment youth (ENTR-only and YRI+ENTR) was selected to participate in a post-YRI follow-up assessment. Fundraising is underway to marshal resources to examine longer-term effects of the YRI, including economic impacts, as integrated into this alternate delivery platform.

### Sample

The feasibility pilot was undertaken in two chiefdoms of Kailahun district, with two gender-segregated clusters per chiefdom. The pilot included a total of 175 youth (62% female, ages 18–30), 120 third-party reporters, 16 facilitators, 4 YRI experts, and two agency leaders. The scale-out study involved 1151 youth (47% female, ages 18–30), 618 third-party reporters, 12 YRI facilitators, and two agency stakeholders for a total sample size of 1783 participants across sites. In both the pilot and scale-out study, youth were eligible if they met study screening criteria which included elevated *t*-scores on assessments of functional impairment and emotional dysregulation, as measured by the WHO Disability Assessment Schedule (WHODAS) [22] and the Difficulties in Emotion Regulation Scale (DERS) [23], respectively. Youth also had to reside in our catchment area, be disengaged from school and formal employment, and for female youth, could not be pregnant given the higher-level support that would be required.

### Data collection

#### Pilot

Given timing constraints and funding limitations, quantitative dissemination and implementation (D&I) surveys were not administered. Rather, scheduled qualitative interviews were restructured to include important implementation science domains. Sixteen YRI facilitators, four YRI experts, and two agency leaders participated in key informant interviews (See Table 2 for a summary) that assessed challenges and successes of the YRI and ENTR, facilitator preparation, perceptions regarding the YRI training, delivery, supervision, and its appropriateness in meeting participant needs and facilitators’ professional goals. The seed team completed a self-report quantitative assessment battery to assess cohesion, collaboration, and leadership [24–27]. Ongoing monitoring was conducted throughout the intervention delivery. The YRI facilitators documented youth attendance for each YRI session. To access YRI facilitator adherence to the intervention structure, sessions were observed in person by a seed team member or audio-recorded. When observing or listening, the seed team members evaluated YRI facilitators’ fidelity to evidence-based YRI practices using a fidelity monitoring checklist, informed by previous interventions using evidence-based components [28]. The pilot data were then used to refine quantitative and qualitative measures administered in the larger scale-out study.

**Table 2:** Implementation science domains examples/quotes captured in qualitative interviews with YRI facilitators

**Acceptability**	
▪ “[The youth] have confessed that they really need this.” (MBF1) ▪ “Those who are attending the YRI, they are, they really been helped with kind of managing their anger, things that has to do with violence.” (MBF4) ▪ “Because I am seeing it, the testimonies, with these guys that I am training, how it has changed their lives, how it has mold their lives.” (MKF5) ▪ “It's made a huge impact in the life of the participants we are dealing with presently....So by the time you are done with them 6 weeks, all of them, their mindsets will change. Their perspective about life will change. And you'll notice those people were going around to be change agents because we don't want to just train them and leave them. They need to be change agents in their communities.” (FBF2) ▪ “And also for them, because before we set this, we asked their consent, ‘Would you be okay with this time?’ And they are the ones that gave us the time that we are dealing now” (FBF1)	
**Adoption**	
**By the participants:** ▪ “Some of them are even practicing it now at home... If you leave them now, you come after a day or tomorrow, you ask them to explain how they use a particular activities, how they use those techniques those skills to calm down their situation.” (MKF5) ▪ “Even when we meet in the street they just tell me, ‘Oh I just notice something about that pot of boiling water and I used this skill that you taught me. I used stop, think, and speak. I used fun activities to interrupt my bad mood that I had’. So that alone is a success for me.” (MBF1) **By the facilitators:** ▪ “Not just the participants now, but even with the facilitators, most of us, we are transformed.” (MKF6) ▪ “After the training, in fact, we have, we began to use the words, the safe place, we began to use the rock in the shoe, even the parables. We began to use all these words now, like um connecting them to ourselves and anything we want to say, we began to, we began to use the words we've been used in the training … The more I keep talking to them, I'm also talking to myself. The more we keep facilitating training them, we're also training ourselves.” (FBF2) ▪ “I take myself as a case study. I was, in that emotional stress before, but since the sessions and the facilitators we’re using its somehow useful to me.” (MBF2). ▪ “And the training helps me how to manage my anger, you know, when I'm angry, things that I shouldn't do and things that I should do to calm myself … The training have helped me to control my feelings.” (MKF4) ▪ “I really hope that the same thing that I received for the training, my participants also receive.” (FBF1)	
**Appropriateness**	
▪ “They got the parables so well and for our participants up to this point, like I don't think there's been any parable that they are finding difficulty to explain in a different way because it is so clear. So and it was surprising to me.” (MBF4) ▪ “Well some of the things were like the parables. When I look at the sessions, I look at the parables, they are like educative, especially when the parables are given in the dialect that everybody can understand in Krio.” (MKF6) ▪ “They are people that the really need what we are taught, who really need to be taught about these things. How to change their feelings, how to change the way they are behaving, the traumas they have, the past things that happens to them, which they think they have lost everything they are nobody. So we learnt how to bring these people back.” (FKF2)	
**Feasibility**	
**Skills:** ▪ “I was really prepared to come and give this message to these people that I was taught over there. Because I have an impact.” (FKF2) ▪ “Challenges are sure to come, they are inevitable. But if you get prepared for them, you can overcome them. So I'm very prepared for any challenge to come my way because I know I have gone through the manual and even if I have a problem with any of the topics in the manual I will know how to tackle that particular problem.” (MBF1) ▪ “So for doing that over and over again for two weeks, it really helped us. So the training became part of us. So now you feel ready, you feel prepared to deliver it to the youth.” MBF4) ▪ “So I'm very prepared for any challenge to come my way because I know I have gone through the manual and even if I have a problem with any of the topics in the manual I will know how to tackle that particular problems” (MBF1) **Time:** ▪ “The time is okay. The 90 minutes will be exceeded at times but you won't exceed more than 10 minutes. The 90 minutes duration is okay. There are times in sessions when you are just below the 90 minutes a bit. And also the number of these per week is ok. Because some of these guys are students, some of them are teachers so we are just working with their time, so it's okay. Yeah, it's good for us.” (MKF5) **Resources:** ▪ “Well everything went perfectly because even when we are here we don't strain or struggle for anything. They always give us what we have a per diem, food, the lodging.” (FKF2) ▪ “Thinking of accommodation, transportation, this, that, everything's okay. [The] salary was surprising, because it was more than the way I was expecting and I believe then we were expecting” (MKF4)	
**Scale/Spread**	
▪ “Every youth we that have got this training, out of 100 percent, 90 percent of them are telling us that they are also conducting the YRIs. So it's like when they learn one, that single individual, will also impart the knowledge to more than two, three or four people … I just hope that such training with continue to thousands of Sierra Leoneans because I believe that if one third of Sierra Leoneans got such training believe me life would change.” (MKF4) ▪ “The models we are using them they are using it to teach other people. So I really changed their life” (FKF2) ▪ “Because we need this type of training. We have gone through a lot, considering the war, the Ebola, the mudslides so there are many people out there that needs these trainings. Whether you are farmer or a business man, this particular training is very important for every Sierra Leonean, it's important … If it's organized on the radio, it would be nice so everyone has access to it.” (MKF5) ▪ “They say wow, this program is good. And they want to take this program to the radio station.” (MKF2) ▪ “[The youth] are even proposing to us that we spread it across the entire country.” (MBF1) ▪ “It important that the YRI is being extended to other communities, other districts.” (MKF6) ▪ “One of things will do is like to have more young people been trained, to have more young people trained as facilitators as well as supervisors. So when once more people are trained as facilitators and supervisors, you will notice that the message will actually spread.” (MKF6)	
**Reach/Access**	
▪ “I think that this message that I heard over there will be also important to these people here.” (FKF2) ▪ “The youth readiness intervention is presently dealing with the feelings, the emotions. Right? And when you look around 90-95 percent of our youth are going through these feelings and emotions.” (FBF2)	
**Fidelity**	
**Supervision and fidelity monitoring** ▪ “We can go out as facilitators, we can do our job. But many a times, if people go out without supervisors they will tend to do otherwise, you know. But if there is a supervisor there, they will be helping them, coming around. Then they know there is somebody on top that is watching them, you know following them.” (FBF2) ▪ “We go through the handouts before going and also we have session debriefing, you know. But otherwise, naturally we can deliver. We can deliver. It's just going through them, there were some things will escape us and the supervisors will like help us” (FBF2) ▪ “Because if we didn't have the supervisors out there, only rely on what we were taught in Freetown, we are human. We are bound to forget. So, the supervision here is good because it keeps us on our toes and it has been a lot to us that keeps our memory fresh for the next day, for proper delivery.” (FKF4) ▪ “If the supervisor is not there to correct you, you think oh, you're Mr. Right, everything that you see is right. So no, the YRI would not be successful, but [with] the supervisor, the YRI will be successful.” (MBF2) ▪ “The goal of supervision is to see that the facilitators deliver the right sure. Is to make sure that the facilitators are on the right track. That they should not forget what to deliver and how to deliver it.” (FKF4) ▪ “The goal {of supervision] is to make sure that you deliver exactly what you were trained to do.” (MKF5)	
**Organizational climate**	
▪ “So I felt at home, felt I've met a family … I believe after the YRI training, after this intervention, we will continue as a family.” (FBF2) ▪ “Initially, when we started the training, it was like everybody was new. I had no friend there. Only my colleagues from my same organization. But as the time goes on … I was like, ‘Wow, this shows that I have a very big family and I'm so proud of that’” (MBF1) ▪ “Also from the training I learned how to build a good cohesion. Because working with people from different organizations, well it's very good, and it has really helped me because they share their own knowledge and things so that really helped me a lot. And working with people that are not my age has really helped me because of the experience they've had over the years.” (MBF1) ▪ “We are all from different organizations. I enjoyed the partnerships so much. That you see somebody from Restless Development being paired with somebody from Caritas, somebody from Caritas being paired with somebody from BRAC. There is that coordination. There is that collaboration. So I enjoyed that most” (MKF6).	
**Leadership**	
▪ “I mean our supervisors now, they are very good. They like go really in depth because they understand what the YRI really means and all of the contents in the manual.” (MBF1) ▪ "My supervisor facilitates my welfare. Supposing, let's say, there is something that I should have from either Caritas or GIZ or whoever that I don't. I ensure I report back to him so he will facilitate as to how I will get it and that is happening correctly” (MKF6)	

#### Scale-out

Youth FORWARD includes partnerships across a range of stakeholders in policy and investment, service delivery, research, and capacity building. As such, scale-out study data were collected to assess multi-level stakeholder engagement, implementation process and impact, and clinical effectiveness with 754 youth, 17 facilitators, and two agency leaders (See Table 1 for scale-out study measures and Table 3 for implementation science domain measures).

**Table 3:** Implementation science domain measures

Domain	Consumer (youth participant)	Provider (YRI facilitator)	Organization (agency leader)
**Acceptability**	**◆**	**◆**	**◆**
**Adoption**	**◆**	**◆**	**◆**
**Appropriateness**	**◆**	**◆**	**◆**
**Feasibility**	**◆**	**◆**	**◆**
**Reach/access**	**◆**	**◆**	**◆**
**Fidelity**		**◆**	
**Organizational climate**		**◆**	**◆**
**Leadership**		**◆**	**◆**
**Sustainment**			**◆**
**Implementation cost**	**◆**	**◆**	**◆**

Qualitative data collected at multiple stakeholder levels assessed intervention satisfaction and acceptability, YRI relevance and impact, barriers to participation, and sustainability. Key informant interviews were done with a sample of youth (*n*=90, post-ENTR), YRI facilitators (*n*=17, baseline and post-ENTR), and agency leaders (*n*=2, baseline and post-ENTR).

A random subsample of 400 participants from the YRI+ENTR and the ENTR-only groups completed a reduced quantitative assessment battery just after completion of the YRI, to assess the effectiveness of the YRI training. Third-party reporters (peers, community members, work supervisors, ENTR facilitators) completed a quantitative assessment battery to evaluate changes in youth functioning at baseline (*n*=550), post-YRI (*n*=626), and post-ENTR (*n*=18, administered to ENTR facilitators only). Feasibility of the delivery approach and impact on sustainment of quality in the delivery of the YRI was assessed by the D&I survey to evaluate the core implementation science domains of adoption, acceptability, appropriateness, feasibility, reach/access, organizational climate, leadership in implementing, general leadership skills, and perceived sustainability [29]. The survey was administered to youth assigned to the treatment arms at baseline (*n*=754) and post-ENTR (*n*=744), to the YRI facilitators at baseline (*n*=17) and post-ENTR (*n*=17), and to the agency leaders at baseline (*n*=2) and post-ENTR (*n*=2). During the scale-out study, a challenge emerged when the implementing partner expressed concern over the intent of the D&I measures. Given the competitive nature of contracts in Sierra Leone, questions regarding organizational climate and leadership were viewed as potentially jeopardizing future contracting opportunities. Several rounds of sensitization about the intent and use of the questions needed to occur before the D&I measures could be implemented.

The seed team completed the self-report quantitative assessment battery post-ENTR (*n*=3), which included a Perceived Cohesion Scale, Research Collaboration Survey, Levels of Collaboration Scale, and Seed team assessment questionnaire (see Table 4 for descriptive statistics) [24–27]. They also participated in a focus group discussion that included topics such as implementing seed team structure during YRI delivery, challenges, successes, experiences with supervision, teamwork, and cohesion.

**Table 4:** Seed team descriptive statistics

Scale	Obs.	Mean	SD	Range	α	Av. Inter-item cov.
Perceived Cohesion Scale	3	22.67	1.53	21-24	0.86	0.22
Seed Team Assessment Questionnaire	2	191	28.28	171-211	0.98	0.31
Research Collaboration Scale	3	4.06	0.69	3.30-4.65	0.94	0.68
Levels of Collaboration Scale	3	2.89	0.51	2.33-3.33	0.96	1.94

The same monitoring procedures were applied for the scale-out intervention delivery. Youth attendance was documented, and the seed team members observed or audio-recorded the YRI sessions and then completed the fidelity monitoring checklists.

#### Data analysis

Seed team focuses on group discussions, notes from supervision sessions with facilitators, and agency, facilitator, and supervision key informant interviews were reviewed and coded inductively by the research team using MAXQDA [30]. The seed team utilized audio recordings of YRI delivery in its pilot phase and directly observed sessions in the scale-out to assess fidelity. In the pilot, only nine of the 12 sessions were recorded and available for fidelity monitoring due to technical challenges.

Due to the small sample size, descriptive statistics were used to convey results of facilitator and agency leader D&I surveys and the seed team battery. Multi-level mixed linear effects models were used to predict the effect of different districts on D&I outcomes, controlling for gender and age of youth.

## Results

### Pilot study data

Data collected during the pilot phase of Youth FORWARD’s scale-out into GIZ’s youth employment scheme demonstrated the feasibility and appropriateness of using a CTA to deliver an evidence-based mental health intervention like the YRI. Decisions and adaptations made throughout the scale-out process were guided by findings from pilot data.

Qualitative data from the pilot revealed insights on challenges and successes surrounding youth employment and psycho-social programming in the country (manuscript submitted for publication, Desrosiers et. al.). Facilitator surveys revealed a number of implementation challenges while delivering the YRI during the pilot (See Table 5). For example, facilitators described issues with providing youth with their travel allowance upfront “because if you give them all the money, they [participants] would just go and never come back” (male facilitator). Facilitators also explained an additional consequence of giving the allowance upfront; non-participants often showed up posing as participants, only to accept the travel allowance and disappear. Others faced delays due to participants arriving late or not coming at all. Many requested more time dedicated to training and to delivering each individual module. Finally, facilitators described language and educational barriers for youth, as a result of operating in rural, remote parts of Sierra Leone (manuscript submitted for publication, Desrosiers et al.).

**Table 5:** Challenges and limitations captured in qualitative interviews with YRI facilitators

Challenge	Number (%) facilitators who mentioned challenge	Examples/quotes
**Giving the travel allowance upfront**	7 (44%)	▪ “Because if you give them all the money, they would just go and never come back.” (MBF1) ▪ “We give them the money on the very first day of the session. Some of them, they are only interested in the money. When they collected the money, some of them like you will never see them again. Or one, two or three sessions.” (FBF1)
**Non-participants posing as participants**	8 (50%)	▪ “People take other people's name.” (FKF2) ▪ “We found it very difficult is because a single name, three or four participants will come and answer to it.” (MBF2) ▪ “When you are there, I'm called Rosie, you're called Rosie. As a facilitator, how am I able to distinguish these two Rosies … It sometimes creates a big headache … At the end of the day, there will be people that come that don't have their training or their transport ticket because other people have benefited.” (FKF4)
**Participants arriving late**	5 (31%)	▪ “The greatest challenge for us is with time. You know, like because we are supposed to start our meetings at 9:00 and sometimes they wouldn't be there up to nine thirty, nine thirty-five. Sometimes we even start at 10:00.” (MBF4) ▪ “We don't even start on time. Maybe if we have session at nine, maybe it will be eleven, twelve before even they start to come.” (FBF1)
**Participants not attending**	7 (44%)	▪ “Sometimes we'll go, we'll have one person absent today, another one absent tomorrow.” (MKF6) ▪ “Some we find out they don't come as a result of the distance. That's another challenge. Some are coming from far villages to meet the session. So some because of the distance, they tend not to come.” (MKF6)
**Training is too short**	5 (31%)	▪ “Two weeks for twelve sessions is not enough, really. It is not enough. Because some of us are slow learners and you will not capture quick until second or third day. So the two weeks is not enough.” (MBF4) ▪ “Extend the time of training. Instead of two weeks, at least to prolong it to three weeks or so.” (MKF3)
**Time to deliver modules is too short**	9 (56%)	▪ “Ninety minutes is not enough to cover all this issue. Not so that everyone can understand.” (MKF4) ▪ “It will also be good if the time has been increased to like two hours instead of one hour thirty minutes.” (MKF6)
**Supervisors stretched thin**	9 (56%)	▪ “Supervising two sets of groups which is very, very difficult. So if you increase the number of supervisors I think the YRI will go on very smoothly.” (MBF1) ▪ “I don't think it's helpful because Unisa will be supervising one team whilst the two teams are doing sessions and he will not know whether they will deliver that session well.” (MKF3)
**Language & educational barriers to understanding**	8 (50%)	▪ “Some of them can't understand Krio at all.” (FBF1) ▪ “Especially those that didn't go to school, you know, and some of them, they will understand but they don't know how to speak the Krio.” (FBF2) ▪ “It's not easy to teach someone that has never gone to school. You have to teach her like a baby. You have to say the thing over and over and over and over again.” (FKF4).

Nonetheless, the uptake of the YRI by participants is demonstrated in the attendance records and was well-documented in qualitative interviews with YRI facilitators conducted at the conclusion of the pilot. During interviews, facilitators provided many examples of youth using YRI activities and strategies to solve everyday problems, providing evidence for the youth’s engagement with the YRI and the success of the intervention. Many YRI facilitators were also using the intervention to improve their own lives (manuscript submitted for publication, Desrosiers et al.).

Interviews with the YRI facilitators indicated that the YRI was considered culturally appropriate and relevant (manuscript submitted for publication, Desrosiers et al.). Facilitators spoke to the inner context of the YRI: training and supervision, embeddedness in local communities, and their perceptions regarding fit, relevance, and compatibility of the YRI+ENTR.

### Adaptations

The scale-out study utilized a staggered approach to rolling out the YRI across study districts, which allowed for cost-effective resource sharing between study sites, cross-site learning between CTA stakeholders, and quality improvement through PDSA cycles [31].

As the YRI was scaled out, the CTA addressed initial logistical challenges identified in the pilot study. As facilitators were often not familiar with languages spoken by youth in rural areas of the country, peer translators were utilized in sessions with youth who did not speak Krio. Further, youth were provided with the transportation stiped at the end of the intervention rather than up-front.

As the scale-out study was capable of reaching more rural communities through the partnership with GIZ and their presence throughout Sierra Leone, the CTA structure was challenged to respond to emerging issues in a remote context. The supervision of facilitators across districts was originally intended to be done in-person by the seed team who were themselves having weekly teleconferences with the other CTA members to discuss progress and challenges. However, transportation was difficult or impossible in some areas of the country and the seed team was unable to travel between the YRI training sites as frequently as they intended. Thus, seed team members divided themselves amongst project sites and were hosted in a single community throughout the duration of YRI delivery. CTA Group meetings with the seed team and YRI facilitators shifted to phone calls, and weekly CTA meetings shifted to virtual meetings via Zoom. Poor network connectivity, limited service, and technology literacy made virtual knowledge-sharing challenging at times.

### Scale out study data

The outer contexts, including contextual limitations, macro-level regulation, and intervention logistics and fidelity, were identified as challenges and barriers to YRI delivery and scale-out. Sierra Leone grapples with a health system overtaxed by war, disease outbreaks, and natural disasters, weak governance structures and limited policy supports, a fractured funding environment, prolonged elections that threaten project implementation, and a fragile context that contributes to reticence from donors.

Worsening intervention fidelity over time is a common obstacle to overall effectiveness. Traditionally, implementation models rely heavily on remote expertise and do not prioritize building local capacity. Organizations in Sierra Leone experience high levels of staff turnover without consistent funding to provide employees with long-term employment contracts. One facilitator noted: “My organization told us that [the job is a] 5-month contract. And when we came … all of a sudden they said six weeks so I was not okay” (male facilitator). This culture is not conducive to maintaining institutional knowledge or sustaining intervention delivery over time. Agency leaders at local organizations confirmed challenges in retaining newly trained staff when projects were short-term, citing funding structures as the chief barrier (Program Manager, Caritas). When delivering a program like Youth FORWARD that utilizes a CTA, considerable time and resources must be spent developing strategies to overcome this challenge.

A major challenge arose in 2017 when the elections resulted in a new government, creating uncertainty with government engagement and a smooth transition of power. As the incoming administration appointed new leadership, relationships initially built when designing the study had to be rebuilt. Another challenge arose when the World Bank, Youth FORWARD’s original partner, changed leadership and set back their Youth Employment Program by 2 years for re-design. As a result, PIs and the Scale-Out Study Team had to identify an alternative implementation partner. The team networked across multiple sectors and eventually partnered with GIZ. While GIZ’s programming provides a strong platform from which to deliver the YRI, a partnership with GIZ presented new challenges. Other adaptations were necessary as GIZ rolled out their employment programming, including the contracting delays and unanticipated acceleration of GIZ’s timeline during study scale-out. Overall, understanding and incorporating the Youth FORWARD research activities within GIZ’s more business-oriented style of operating has required ongoing negotiation and adaptations with implications for implementation research.

Youth FORWARD is part of a network of hubs delivering interventions in low-resource settings [31]. Tremendous challenges lie in complying with regulatory processes, sometimes at odds with field realities. Current project oversight mimicked regulatory processes utilized in drug trials and relied on an external and independent Data and Safety Monitoring Board with a fixed meeting schedule and routine study monitoring from a contracted clinical research associate. This level of oversight was in place to ensure study compliance and participant safety but led to study delays given the fixed nature of the review and approval processes, which often did not align with the service delivery requirements of implementation partners. As a result, the need to align study procedures across several ethical review bodies resulted in increased study costs and implementation delays.

Interviews with the YRI facilitators and seed team members also revealed facilitators to implementation and successes of the intervention. Youth councils established by the Sierra Leonean government in 2012 are present in each district, with councils spanning from the district to the village level. These councils are positioned to work across relevant stakeholder groups to amplify youth presence in the policy making process, elevate youth perspectives, and illuminate challenges facing youth in Sierra Leone [32]. As part of its youth employment platform, GIZ works closely with youth councils to support program coordination and implementation. Leveraging this established relationship was an asset for the YRI team given a limited presence in the more rural study districts. As a result, YRI experts and study research assistants relied heavily on youth council leadership to engage, identify, and contact youth. These councils represent an important innovation factor that supported YRI and ENTR implementation with the potential to influence long-term sustainability [33].

The CTA provided a bridging structure that allowed researchers from Boston College and the University of Georgia to learn from local stakeholders with significant programming expertise who are embedded in Sierra Leonean communities, which will contribute to long-term sustainability of the YRI. In addition, the CTA provided opportunities for Sierra Leonean agency members to learn from each other’s experience in mental health and youth programming. One seed team member appreciated how the CTA provided specific implementation guidance: “(The CTA) gives the team a road map to best practice. Clarity within the organization leaves less for assumption and allows all partners involved … to make the best decisions and strengthen the core of the program” (Male seed team member).

Two interviewed agency leaders expressed that the CTA allowed for sharing of knowledge and benefitting from the expertise of each member. As one agency leader described, “one organization might be an expert in one thing and the other might be an expert in the other thing. We’re able to kind of meet and discuss things and strengthen that model that we want to use for greater achievement in the communities. And that seems to be working really well” (Senior Program Manager, Restless Development). During regular meetings, both agency leaders appreciated how members across teams would work together to problem solve as issues arose during implementation. Communication and mission alignment improved throughout the process. Both agency leaders explained how multiple budgets and modes of operation were challenging at first, but ultimately led to increased collaboration. According to one agency leader, “it’s (the CTA) has given us a lot more insight to be able to have more team players in a small pitch. So that we, we coordinate more and, and we share a lot more information...which has been very good” (Program Manager, Caritas).

In supervision sessions, seed team members that comprised the CTA provided guidance to facilitators in a manner that empowered self-reflection and self-monitoring. One seed team supervisor described how he would begin supervision sessions by asking the facilitator what his own perceptions were on his delivery of the YRI before offering facilitation critiques and advice. This approach allowed for critical thinking and growth while maintaining fidelity of the intervention.

All interviewed facilitators expressed appreciation for supervision and provided examples of how group and individual meetings with supervisors assisted with problem solving and content challenges. One facilitator described her experience with supervision:

It is very helpful. My co-facilitator and I will be busy with other things in the manual, and maybe one session is not well explained. (Our supervisor) will be there to observe and she will tell us that you have to explain this area … she will tell us to probe very well so that the participants will get a better understanding. Supervision empowers you to be a good worker … it will empower you to become very self-sufficient in your job (female facilitator).

Mixed linear effects models revealed no significant differences in acceptability, adoption, appropriateness, feasibility, or reach between implementation districts, while accounting for gender and age of participants. These results indicate that the intervention functioned similarly across districts and suggests the effectiveness of the seed team in training and supervising YRI facilitators. D&I descriptive statistics from facilitator and agency leader surveys revealed that the perceptions of intervention adoption, appropriateness, feasibility, and organizational climate increased from baseline to endline, while acceptability, reach, implementation leadership, and general leadership scores decreased (See Tables 6 and 7 on D&I descriptive statistics).

**Table 6:** Descriptive statistics from D&I facilitator surveys (*N*=17)

D&I outcome	Baseline mean (standard deviation)	Endline mean (standard deviation)
Acceptability	3.878 (0.163)	3.771 (0.297)
Adoption	3.201 (0.663)	3.510 (0.383)
Appropriateness	3.716 (0.270)	3.896 (0.130)
Feasibility	3.333 (0.435)	3.855 (0.161)
Reach	3.675 (0.403)	3.206 (0.428)
Organizational climate	3.422 (0.416)	3.458 (0.628)
Implementation leadership	3.750 (0.281)	3.397 (0.662)
General leadership	3.735 (0.450)	3.285 (0.455)

**Table 7:** Descriptive statistics from D&I agency leader surveys (*N*=2)

D&I outcome	Baseline mean (standard deviation)	Endline mean (standard deviation)
Adoption		3.100 (0.141)
Acceptability		3.600 (0.283)
Appropriateness		3.917 (0.000)
Organizational climate		3.800 (0.189)
Implementation leadership		3.792 (0.295)
General leadership		3.833 (0.236)
Feasibility		3.962 (0.054)
Reach		3.283 (0.165)
Sustainability		2.500 (0.707)

## Discussion

The Government of Sierra Leone recognizes that addressing the lingering effects of trauma on the mental health and functioning of youth is a necessary first step in meeting their core needs. Recent policy describes the fundamental relationship between health and economic self-sufficiency, citing that health affects individual productivity and is a critical input for long-term in-country development [34]. The current administration also identifies the benefits generated in the opposite direction, recognizing individual economic self-sufficiency as a major contributor to positive health outcomes, highlighting the potential for economic development initiatives to address health and psychosocial issues [35].

Increased investment in youth employment programs offers a cost-effective alternate system for delivery of mental health services [35]. Embedding a mental health intervention into youth employment programming can add tremendous value since youth receiving psychosocial training will be better regulated and therefore better able to fully engage in the employment programming, perpetuating a positive feedback loop. By leveraging investments in youth and economic development programs and integrating mental health interventions therein, LMICs with limited mental health care infrastructure and personnel can build new capacity to address the mental health treatment gap. If proven feasible, integrated YRI+ENTR programs have high potential to increase access to evidence-based mental health services for thousands of high-risk youth in Sierra Leone and other post-conflict LMICs.

Future work will require a concerted effort to understand how emerging mental health and psychosocial programming can contribute to the development of longer term and sustainable systems of health and mental health. The outer context and inner context of interventions often change, and factors that bridge those contexts must be taken into account. The EPIS framework is particularly helpful in this regard. The challenges of scale-out and sustainment of mental health interventions are tremendous; especially in LMICs where the availability of specialists for implementation, training, and supervision is limited. However, this study reveals several exciting implementation science innovations useful in fragile settings. First, research and interventions should be contextualized with careful consideration of risk and protective factors across all levels of the social ecology. Second, psychosocial interventions should be based on locally identified needs, rather than externally imposed services or assumptions, identifying priorities through community-based approaches and collaboration with local service providers and community advisory boards. Educational and employment programs should be considered and explored as alternative delivery platforms to effectively deliver mental health services by community-based lay workers, addressing the limited human resource and capacity challenges in post-conflict settings. In addition, it may be that mental health interventions that are embedded within work and other daily activities can optimize outcomes. Finally, efforts to address the mental health treatment gap must include attention to implementation science questions on how to take evidence-based mental health services to scale. Achieving such goals will require innovations addressing limited human resources for health, consideration of incentives, training, supervision, cost, and ongoing professional development for intervention staff, financing, and policy structures, along with strategies for monitoring and improving quality.

### Limitations

An existing pilot study seed team was intended to train and monitor new seed teams in the larger scale-out study, creating a community of practice around the YRI. However, given the need for a competitive bidding process, a single-service provider was chosen for the scale-out study, creating a new cadre of facilitators. As such, the seed team for the scale-out study needed to be reconfigured to allow for the selected service provider to be trained and for supervision to be provided by the CTA, with support from Youth FORWARD leadership. This new seed team included Caritas YRI experts who had previously delivered the YRI and participated in the feasibility pilot, thus retaining the knowledge and skills.

## Conclusions

Data demonstrate that GIZ’s youth employment scheme is an auspicious delivery platform for an evidence-based, mental health intervention in a low-resource, post-conflict setting. In fragile settings where youth face challenges accessing mental health care and livelihoods support, it is critical to develop interventions that can be delivered and maintained by local communities. Lay workers can be equipped to deliver evidence-based interventions when supported by a local seed team of expert facilitators. Finally, our findings provide further evidence that the Collaborative Team Approach promotes local ownership and capacity while supporting quality improvement and sustainability of evidence-based interventions.

## Data Availability

The datasets generated and/or analyzed during the current study are not publicly available due continued analysis by Youth FORWARD researchers but are available from the corresponding author on reasonable request.

## Abbreviations

CTA: Collaborative team approach
DERS: Difficulties in Emotion Regulation
D&I: Dissemination & Implementation
EPIS: Exploration, Preparation, Implementation, and Sustainment
ENTR: Entrepreneurship training
GIZ: Deutsche Gesellschaft für Internationale Zusammenarbeit
LSWAY: Longitudinal Survey of War-Affected Youth
LMIC: Low- and middle-income country
NIH: National Institute of Health
PDSA: Plan-Do-Study-Act
PI: Principal investigator
RCT: Randomized control trial
RPCA: Research Program on Children and Adversity
WHO: World Health Organization
WHODAS: World Health Organization Disability Adjustment Scale
Youth FORWARD: Youth Functioning and Organizational Success for West African Regional Development
YRI: Youth Readiness Intervention

## References

[1] Charlson F, Ommeren M v, Flaxman A, Cornett J, Whiteford H, Saxena S (2019). New WHO prevalence estimates of mental disorders in conflict settings: a systematic review and meta-analysis. Lancet..

[2] Betancourt TS, Brennan RT, Rubin-Smith J (2010). Sierra Leone's former child soldiers: a longitudinal study of risk, protective factors, and mental health. J Am Acad Child Adolesc Psychiatry..

[3] Betancourt TS, McBain R, Newnham EA, Brennan RT (2013). Trajectories of internalizing problems in war-affected Sierra Leonean youth: examining conflict and post conflict factors. Child Dev.

[4] Betancourt TS, Borisova II, de la Soudière M, Williamson J (2011). Sierra Leone's child soldiers: war exposures and mental health problems by gender. J Adolesc Health.

[5] Sharma M, Fine SL, Brennan RT, Betancourt TS. Coping and mental health outcomes among Sierra Leonean war-affected youth: results from a longitudinal study. Dev Psychopathol. 2016;(1):1–13. 10.1017/S0954579416001073 27866500

[6] JRI Research and Training Institute, Inc. Strengthening access to mental health services in Sierra Leone. https://publications.jsi.com/JSIInternet/Inc/Common/_download_pub.cfm?id=19144&lid=3 Accessed 19 Dec 2020.

[7] International Monetary Fund. Sierra Leone Economic Development Documents: National Development Plan 2019-23. https://www.imf.org/en/Publications/CR/Issues/2019/07/09/Sierra-Leone-Economic-Development-Documents-National-Development-Plan-2019-23-47099. Accessed 22 Dec 2020.

[8] World Bank Group Launches New Country Partnership Framework for Sierra Leone. https://www.worldbank.org/en/news/press-release/2020/06/05/world-bank-group-launches-new-country-partnership-framework-for-sierra-leone. Accessed 22 Dec 2020.

[9] GIZ. Employment promotion programme – needs oriented qualification for youth. Factsheet. https://www.giz.de/en/downloads/giz-Employment%20Prom-Progr%20Sierra-leone_191217_web.pdf Accessed 19 Dec 2020.

[10] GIZ. Employment Promotion Programme (EPP III) Youth Development. https://www.giz.de/en/downloads/flyer%20capacity%20building%20for%20youth-WEB.pdf. Accessed 19 Dec 2020.

[11] Betancourt T, Newnham E, Hann K, Raynaud J, Gau S, Hodes M (2014). Addressing the consequences of violence and adversity: the development of a group mental health intervention for war-affected youth in Sierra Leone. From Research to Practice in Child and Adolescent Mental Health.

[12] Newnham EA, McBain RK, Hann K, Akinsulure-Smith AM, Weisz J, Lilienthal GM (2015). The Youth Readiness Intervention for war-affected youth. J Adolesc Health..

[13] Betancourt TS, McBain R, Newnham EA (2014). A behavioral intervention for war-affected youth in Sierra Leone: a randomized controlled trial. J Am Acad Child Adolesc Psychiatry.

[14] Ogrinc G, Davies L, Goodman D, Batalden PB, Davidoff F, Stevens D (2016). SQUIRE 2.0 (Standards for QUality Improvement Reporting Excellence): Revised publication guidelines from a detailed consensus process. BMJ Qual Safety..

[15] Curran GM, Bauer M, Mittman B, Pyne JM, Stetler C (2012). Effectiveness-implementation hybrid designs: combining elements of clinical effectiveness and implementation research to enhance public health impact. Medical care..

[16] Moullin JC, Dickson KS, Stadnick NA, Rabin B, Aarons GA (2019). Systematic review of the Exploration, Preparation, Implementation, Sustainment (EPIS) framework. Implementation Sci..

[17] Stadnick NA, Brookman-Frazee L, Mandell DS, Kuelbs CL, Coleman KJ, Sahms T (2019). A mixed methods study to adapt and implement integrated mental healthcare for children with autism spectrum disorder. Pilot Feasibility Study..

[18] Institute for Healthcare Improvement (2017). Quality improvement essentials toolkit.

[19] Franzen SRP, Chandler C, Lang T (2017). Health research capacity development in low and middle income countries: reality or rhetoric? A systematic meta-narrative review of the qualitative literature. BMJ Open..

[20] Aarons G, Fettes D, Hurlburt M (2014). Collaboration, negotiation, and coalescence for interagency-collaborative teams to scale-up evidence-based practice. J Clin Child Adolesc Psychol.

[21] Hurlburt M, Aarons GA, Fettes D, Willging C, Gunderson L, Chaffin MJ (2014). Interagency Collaborative Team model for capacity building to scale-up evidence-based practice. Children Youth Services Rev.

[22] ÜstÜn TB (2010). World Health Organization. Measuring health and disability: manual for WHO Disability Assessment Schedule WHODAS 2.0.

[23] Gratz KL, Roemer L (2004). Multidimensional assessment of emotion regulation and dysregulation: development, factor structure, and initial validation of the Difficulties in Emotion Regulation Scale. J Psychopathol Behav Assess.

[24] Chin WW, WmD S, Pearson AW, Stollak MJ (1999). Perceived cohesion in small groups: adapting and testing the perceived cohesion scale in a small-group setting. Small Group Res.

[25] Seed Team Assessment Questionnaire. Content last reviewed. Agency for Healthcare Research and Quality. Rockville; 2012. http://www.ahrq.gov/teamstepps/instructor/reference/tmassess.html

[26] Mâsse LC, Moser RP, Stokols D, Taylor BK, Marcus SE, Morgan GD (2008). Measuring collaboration and transdisciplinary integration in team science. Am J Prev Med..

[27] Frey BB, Lohmeier JH, Lee SW, Tollefson N (2006). Measuring collaboration among grant partners. Am J Eval.

[28] Ward AM, Regan J, Chorpita BF, Starace N, Rodriguez A, Okamura K, Daleiden EL, Bearman SK, Weisz JR (2013). Research Network On Youth Mental Health. Tracking evidence based practice with youth: validity of the MATCH and standard manual consultation records. J Clin Child Adolesc Psychol..

[29] Haroz EE, Bolton P, Nguyen AJ, Lee C, Bogdanov S, Bass J, Singh NS, Doty SB, Murray L (2019). Measuring implementation in global mental health: validation of a pragmatic implementation science measure in eastern Ukraine using an experimental vignette design. BMC health services research..

[30] Software VERBI (2017). MAXQDA 2018 [computer software].

[31] Betancourt TS, Hansen N, Farrar J, Borg RC, Callands T, Desrosiers A (2020). Youth functioning and organizational success for West African Regional Development (Youth FORWARD): Study Protocol. PS.

[32] Ministry of Youth Affairs. A blueprint for youth development Sierra Leone’s National Youth Programme. 2014-2018. 2014. http://nationalyouthcommission.sl/pdf%20files/blue%20print.pdf. Accessed 1 Feb 2022.

[33] Lengnick-Hall R, Willging C, Hurlburt M, Fenwick K, Aarons GA (2020). Contracting as a bridging factor linking outer and inner contexts during EBP implementation and sustainment: a prospective study across multiple U.S. public sector service systems. Implementation Sci..

[34] Government of Sierra Leone Ministry of Health and Sanitation. National Health Sector Strategic Plan. 2010-2015. November 2009. http://www.ministerial-leadership.org/sites/default/files/resources_and_tools/Abridged%20NHSSP.pdf. Accessed 19 Dec 2020.

[35] National Youth Commission, Ministry of Youth Employment and Sports. Youth Development: Sierra Leone Youth Report. 2012. http://www.sl.undp.org/content/dam/sierraleone/docs/projectdocuments/povreduction/sl_status_ofthe_youth_report2012FINAL.pdf. Accessed 19 Dec 2020.

